# Metformin as a disease-modifying therapy in osteoarthritis: bridging metabolism and joint health

**DOI:** 10.3389/fphar.2025.1567544

**Published:** 2025-03-19

**Authors:** Iryna Halabitska, Pavlo Petakh, Oleksandr Kamyshnyi

**Affiliations:** ^1^ Department of Therapy and Family Medicine, I. Horbachevsky Ternopil National Medical University, Ternopil, Ukraine; ^2^ Department of Biochemistry and Pharmacology, Uzhhorod National University, Uzhhorod, Ukraine; ^3^ Department of Microbiology, Virology, and Immunology, I. Horbachevsky Ternopil National Medical University, Ternopil, Ukraine

**Keywords:** osteoarthritis, impaired glucose tolerance, metformin, inflammatory markers, disease-modifying therapy

## Abstract

**Background:**

Osteoarthritis (OA) and impaired glucose tolerance (IGT) frequently coexist, leading to compounded clinical and metabolic challenges. This study investigates the effects of metformin in improving both clinical outcomes (pain, stiffness, physical function) and metabolic parameters (inflammatory markers, lipid profile, BMI) in patients with knee OA and IGT.

**Methods:**

The study included 60 patients diagnosed with knee OA and IGT. Participants were divided into two groups: 26 patients received standard OA treatment without metformin (Without Metf), while 34 received metformin (500 mg twice daily) for 3 months, in addition to standard treatment (With Metf). Clinical assessments (WOMAC, Lequesne Algofunctional Index, KOOS, VAS) and metabolic markers (CRP, NLR, SOD, lipid profile, BMI) were measured before treatment, after 1 month, and after 3 months.

**Results:**

The With Metf group showed significantly greater improvements in pain, stiffness, physical function, and quality of life compared to the Without Metf group. Metformin also led to significant reductions in inflammatory markers and improvements in lipid profiles and metabolic health indicators. The With Metf group demonstrated enhanced BMI, waist-to-hip ratio, and waist-to-height ratio. Furthermore, the need for increased NSAID doses was predicted by factors such as pain severity and inflammatory markers.

**Conclusion:**

Metformin effectively alleviates osteoarthritis symptoms and improves metabolic health in patients with both OA and IGT. Further research is needed to explore its long-term effects on joint health, inflammatory markers, and its potential role in OA management in patients without IGT.

## 1 Introduction

The investigation of pleiotropic drugs in osteoarthritis (OA) is focused on identifying agents that simultaneously reduce symptoms and target underlying pathogenic mechanisms, potentially slowing disease progression (Fazio et al., 2024; Coppola et al., 2024; Halabitska et al., 2024a). Metformin, a first-line pharmacological agent widely used for managing type 2 diabetes mellitus (T2DM), has garnered considerable attention for its effects beyond glycemic control (Baker et al., 2021; Bailey, 2024). As an insulin-sensitizing agent, metformin is also frequently prescribed for individuals with impaired glucose tolerance (IGT), a prediabetic condition characterized by disrupted glucose homeostasis, systemic inflammation, and metabolic dysfunction (Ping et al., 2024; Hostalek et al., 2015; Rojas and Gomes, 2013). While its primary therapeutic role is in improving insulin sensitivity and reducing hepatic gluconeogenesis, growing evidence suggests that metformin exerts pleiotropic effects, including anti-inflammatory, antioxidant, and metabolic regulatory properties (Dutta et al., 2023; Apostolova et al., 2020; Drzewoski and Hanefeld, 2021). These attributes position metformin as a promising candidate for addressing a range of metabolic and degenerative disorders (Rotermund et al., 2018; Isop et al., 2023; Petrie, 2024).

OA, a chronic and progressive degenerative joint disease, has traditionally been associated with mechanical factors such as joint overload and injury (He et al., 2020; Felson, 2013; Hall et al., 2016; Halabitska and Babinets, 2021). OA is one of the most prevalent musculoskeletal disorders worldwide (Allen et al., 2022; Global, 2023). IGT affects a significant portion of the population, particularly in older adults (Kalyani and Egan, 2013; Fang et al., 2019; Hermans et al., 2005). Their comorbidity is common and poses challenges due to overlapping inflammatory and metabolic pathways (Aziz et al., 2024; Berenbaum and Walker, 2020). However, emerging evidence highlights the critical role of metabolic and inflammatory mechanisms in its pathogenesis, particularly in individuals with metabolic comorbidities such as obesity, T2DM, and IGT (Chandrasekaran and Weiskirchen, 2024; Rohm et al., 2022; Ruze et al., 2023; Redkva et al., 2021; Zemlyak et al., 2023). The comorbidity of osteoarthritis and obesity highlights a complex interplay of systemic inflammation and metabolic disturbances, exacerbating the progression of both conditions (Halabitska et al., 2021; Nedunchezhiyan et al., 2022; Halabitska et al., 2024b). These comorbidities create a vicious cycle (Li B. et al., 2024; Swain et al., 2022; Repchuk et al., 2021). This interaction not only accelerates joint degeneration but also contributes to chronic low-grade inflammation, insulin resistance, and impaired overall metabolic homeostasis (Halabitska et al., 2024c; Vinuesa et al., 2021; Roberts et al., 2013). OA is characterized by cartilage degradation, subchondral bone remodeling, and synovial inflammation, processes that are exacerbated by systemic metabolic dysfunction (He et al., 2020; De Roover et al., 2023; Halabitska et al., 2024d). In this context, the metabolic and anti-inflammatory actions of metformin may have a dual benefit: addressing systemic metabolic derangements and modulating local joint pathology (Foretz et al., 2023; Domingo et al., 2024; He, 2020).

Preliminary studies have demonstrated that metformin can mitigate key mechanisms underlying OA progression (Xu et al., 2024; Anis et al., 2012; Li et al., 2020; Lambova, 2023). These include reductions in systemic inflammation and oxidative stress, improvements in lipid metabolism, and direct modulation of chondrocyte function (Adam et al., 2024; Horváth et al., 2023; Su et al., 2022). Moreover, metformin has been shown to enhance the synthesis of extracellular matrix components, promoting cartilage repair and potentially slowing the degenerative processes associated with OA (Feng et al., 2020; Yao et al., 2023; Zheng et al., 2021; Song et al., 2022). Despite these promising findings, the clinical application of metformin in OA remains underexplored, and robust evidence from longitudinal studies and clinical trials is necessary to validate its therapeutic potential.

This article aims to explore the potential role of metformin in managing OA in patients with IGT. By addressing both systemic and local pathological mechanisms, metformin may offer a novel therapeutic approach for patients with these comorbid conditions. Further elucidation of its disease-modifying properties could pave the way for integrating metformin into broader treatment paradigms for OA, particularly in the context of metabolic health optimization.

## 2 Materials and methods

### 2.1 Subjects

The study included 60 patients diagnosed with knee OA and IGT. Inclusion criteria required participants to have a confirmed diagnosis of OA based on the American College of Rheumatology (ACR), EULAR, and National Institute for Health clinical and radiographic criteria (Peat et al., 2006; Wang et al., 2024), which include the presence of pain, stiffness, or functional limitation in at least one joint, along with radiographic evidence of joint space narrowing, osteophytes, and subchondral sclerosis. Additionally, participants had to meet the criteria for IGT, defined by a fasting blood glucose level between 5.6 and 6.9 mmol/L (100–125 mg/dL) or a 2-h postprandial glucose level between 7.8 and 11.0 mmol/L (140–199 mg/dL), in accordance with the World Health Organization (WHO) criteria (Bergman et al., 2024).

Exclusion criteria included the presence of other metabolic or systemic conditions that could confound the results, such as uncontrolled diabetes mellitus, cardiovascular disease, or inflammatory rheumatic diseases. Patients with a history of joint surgery, joint replacement, or other significant comorbidities such as malignancies were also excluded from the study.

The study was conducted in accordance with the core principles outlined in the Council of Europe’s Convention on Human Rights and Biomedicine, as well as the ethical guidelines set forth in the World Medical Association’s Declaration of Helsinki on medical research involving human subjects, including its subsequent revisions (World Medical Association Declaration of Helsinki, 2014). Additionally, the research adhered to the regulations specified in Ministry of Health of Ukraine Order No. 690, dated 23 September 2009. All participants provided written informed consent before their participation. Ethical approval for the study was granted by the Bioethics Committee of I. Horbachevsky Ternopil National Medical University, Ministry of Health of Ukraine (Protocol No. 78, 18 August 2024).

The study cohort was divided into two groups: 26 patients in the Without Metf group and 34 patients in the With Metf group, with matched characteristics in terms of age, gender, severity, and disease progression of osteoarthritis (Table 1). The Without Metf group received OA treatment according to the established protocol, along with recommendations for improving glucose tolerance, including dietary modifications (e.g., reducing carbohydrate intake, increasing dietary fiber, and promoting a balanced nutritional regimen), regular physical exercise, and other lifestyle interventions. The With Metf group received OA treatment in accordance with the protocol, in addition to receiving metformin at a dose of 500 mg twice daily for a period of 3 months. All clinical parameters were assessed before treatment, after 1 month, and after 3 months.

**TABLE 1:** Demographic and OA duration characteristics.

Indicator	Without Metf (n = 26)	With Metf (n = 34)	p-value
Male	57.69%	55.88%	p = 0.432 (MW)
Age	47 (35–52.75)	48 (34–53)	p = 0.279 (MW)
Duration of OA	7 (4–9)	7 (4.5–9)	p = 0.725 (MW)
Kellgren–Lawrence Grade	2 (2–2)	2 (2–2)	p = 0.244 (MW)

Median and interquartile range (IQR) were used to summarize the data, p–Mann-Whitney U-test (MW).

### 2.2 Laboratory and clinical data

The Western Ontario and McMaster Universities Osteoarthritis Index (WOMAC) was employed to evaluate pain, stiffness, physical function, and overall health status in patients with OA. It consists of three subscales: Pain (P), Stiffness (S), and Function (F). An overall score (OS) is calculated based on the combined results from these subscales, offering a holistic assessment of the patient’s condition (Ebrahimzadeh et al., 2014).

The Lequesne Algofunctional Index (LI) was used to assess pain, functional impairment, and overall health status in patients with OA. It consists of two primary components: Pain Assessment (PA) and Functional Impairment Assessment (FIA). An Overall Score (OS) is derived from the combined results of both components, providing a comprehensive evaluation of the patient’s condition (Faucher et al., 2002).

The Knee injury and Osteoarthritis Outcome Score (KOOS) scale was utilized to evaluate various aspects of knee health in patients with osteoarthritis. It includes five subscales: Pain (P), Other Symptoms (OS), Function in Daily Living (FDL), Function in Sport/Recreation (FS/R), and Knee-Related Quality of Life (KRQL). These subscales collectively provide a comprehensive assessment of pain, functionality, and quality of life related to knee osteoarthritis (Roos and Lohmander, 2003).

The Visual Analog Scale (VAS) was employed to assess pain (VAS-P) in patients with osteoarthritis. Additionally, functional limitations (VAS-FL), stiffness (VAS-S), and physical activity and mobility (VAS-PAM) were evaluated to gain a more comprehensive understanding of the impact of osteoarthritis on daily functioning and quality of life (Delgado et al., 2018).

The Timed Up and Go (TUG) test and the 6-Minute Walk Test (6MWT) assessed functional mobility and endurance in patients with OA. The TUG measures the time to rise from a chair, walk a set distance, turn, return, and sit, while the 6MWT evaluates the distance walked in 6 minutes, indicating physical endurance and functional capacity (Montgomery et al., 2020; Buisseret et al., 2020).

The SF-36 Health Survey was used to assess health-related quality of life in patients at three time points: before treatment, after 1 month, and after 3 months. The survey included the following scales: Physical Functioning (SF-36-PF), Role Limitations due to Physical Health (SF-36-RP), Bodily Pain (SF-36-BP), General Health (SF-36-GH), Vitality (SF-36-VT), Social Functioning (SF-36-SF), Role Limitations due to Emotional Health (SF-36-RE), and Mental Health (SF-36-MH) (Ware and Sherbourne, 1992; Ware, 2000).

Anthropometric measurements were taken to assess patients’ metabolic health, including Body Mass Index (BMI), calculated as weight in kilograms divided by height in meters squared (kg/m^2^); Waist-to-Hip Ratio (WHR), determined by dividing waist circumference by hip circumference; and Waist-to-Height Ratio (WHtR), calculated as the ratio of waist circumference to height.

Fasting plasma glucose (FPG) was measured in mmol/L using an enzymatic method on the Cobas c311 analyzer (Roche Diagnostics, Germany); sensitivity: 0.11 mmol/L; measurement range: 0.11–41.7 mmol/L; intra-assay CV <2%; analyzed in duplicate. Glycated hemoglobin (HbA1c) was determined as a percentage (%) by high-performance liquid chromatography (HPLC) using the Tosoh G8 HPLC Analyzer (Tosoh Corporation, Japan); sensitivity: 0.1%; measurement range: 3.0%–18.0%; intra-assay CV <2%; analyzed in duplicate. The Homeostasis Model Assessment of Insulin Resistance (HOMA-IR) was calculated using the formula: HOMA-IR = (FPG × fasting insulin)/22.5. C-peptide levels were measured in ng/mL using an immunoassay method on the Architect i2000SR analyzer (Abbott, USA); sensitivity: 0.02 ng/mL; measurement range: 0.02–20 ng/mL; intra-assay CV <5%; analyzed in duplicate.

The Neutrophil-to-Lymphocyte Ratio (NLR) was calculated by dividing the neutrophil count by the lymphocyte count, both measured in cells/µL both measured in cells/µL using the Sysmex XN-1000 hematology analyzer (Sysmex Corporation, Japan); sensitivity: 0.01 × 10^9^/L; measurement range: neutrophils 0.01–100 × 10^9^/L, lymphocytes 0.01–50 × 10^9^/L; intra-assay CV <3%; analyzed in duplicate. C-Reactive Protein (CRP) was quantified in mg/L using an immunoturbidimetric assay using an immunoturbidimetric assay on the Cobas c501 analyzer (Roche Diagnostics, Germany); sensitivity: 0.3 mg/L; measurement range: 0.3–350 mg/L; intra-assay CV <3%; analyzed in duplicate. Hydroxyproline (HP) levels were determined in mg/L by colorimetric analysis using the Shimadzu UV-1800 spectrophotometer (Shimadzu, Japan); sensitivity: 0.5 mg/L; measurement range: 0.5–100 mg/L; intra-assay CV <5%; analyzed in triplicate. Malondialdehyde (MA) was measured in µmol/L using a thiobarbituric acid reactive substances assay on the Agilent Cary 60 UV-Vis spectrophotometer (Agilent Technologies, United States); sensitivity: 0.1 μmol/L; measurement range: 0.1–25 μmol/L; intra-assay CV <5%; analyzed in triplicate. Superoxide Dismutase (SOD) activity was assessed in U/mL using a spectrophotometric method on the Beckman Coulter DU 730 analyzer (Beckman Coulter, United States); sensitivity: 0.05 U/mL; measurement range: 0.05–25 U/mL; intra-assay CV <5%; analyzed in triplicate. α_1_-Antitrypsin (α_1_-AT) concentrations were measured in g/L by nephelometry on the BN ProSpec analyzer (Siemens Healthineers, Germany); sensitivity: 0.1 g/L; measurement range: 0.1–4.5 g/L; intra-assay CV <3%; analyzed in duplicate.

Total Cholesterol (TC) was measured in mmol/L using a colorimetric enzymatic method on the Abbott Architect c8000 analyzer (Abbott, United States); sensitivity: 0.1 mmol/L; measurement range: 0.1–15.0 mmol/L; intra-assay CV <3%; analyzed in duplicate. Low-Density Lipoprotein (LDL) cholesterol was quantified in mmol/L using the direct measurement method on the Abbott Architect c8000 analyzer (Abbott, United States); sensitivity: 0.2 mmol/L; measurement range: 0.2–10.0 mmol/L; intra-assay CV <3%; analyzed in duplicate. High-Density Lipoprotein (HDL) cholesterol levels were determined in mmol/L by a homogeneous enzymatic assay on the Abbott Architect c8000 analyzer (Abbott, United States); sensitivity: 0.1 mmol/L; measurement range: 0.1–4.0 mmol/L; intra-assay CV <3%; analyzed in duplicate. Triglycerides (TG) were assessed in mmol/L using an enzymatic colorimetric method on the Abbott Architect c8000 analyzer (Abbott, United States); sensitivity: 0.1 mmol/L; measurement range: 0.1–11.3 mmol/L; intra-assay CV <3%; analyzed in duplicate.

The OA exacerbation characteristics were assessed as follows: duration of exacerbation (≤7 days or >7 days) (OA exc. dur.), frequency of exacerbations in the last 3 months (≤2 times or >2 times) (OA exc. freq.), need for additional medical interventions during exacerbation (e.g., injections, physiotherapy) (OA exc. med. int.), requirement for work leave or exemption due to exacerbation (OA exc. work leave), occurrence of accompanying symptoms (e.g., swelling, redness) (OA exc. acc. sym.), need for increasing the dose of NSAIDs (NSAIDs dose), and need for increasing the duration of NSAID use (NSAID dur.).

### 2.3 Statistical analysis

Quantitative variables were first tested for normality using the Shapiro-Wilk test. Variables were described using median (Me) and lower and upper quartiles (Q1 – Q3). Categorical data were described with absolute and relative frequencies. The Mann-Whitney U-test was used to compare two groups on a quantitative variable. The comparison of frequencies in the analysis of 2 by 2 contingency tables was performed using Fisher’s exact test. As a measure of the effect size when comparing groups regarding binary variables, the odds ratio (OR) with a 95% confidence interval (95% CI) was calculated. The Friedman test was used, along with the Conover-Iman test with Holm correction as a *post hoc* method. A prognostic model for the probability of a specific outcome was constructed using logistic regression. The coefficient of determination, indicating the portion of variance explained by the logistic regression, was evaluated using Nagelkerke’s R^2^. For assessing the discriminatory ability of quantitative variables in predicting a specific outcome, ROC curve analysis was performed. The cut-off point for the quantitative variable was determined by the highest value of the Youden index. Differences were considered statistically significant at p < 0.05.

Statistical analyses were conducted using commercially available software packages, including IBM SPSS Statistics (version 25), R (version 4.0.3), and GraphPad Prism (version 9.3). These programs were used for data management, statistical testing, and generating visual representations of the results.

## 3 Results

### 3.1 Analysis of clinical outcomes across treatment groups

In both studied groups, statistically significant differences were observed. The analysis of WOMAC index dynamics revealed significant improvements in the WOMAC-P, WOMAC-S, WOMAC-F, and WOMAC-OS, with notable changes observed both before treatment *versus* after 3 months and after 1 month *versus* after 3 months across all scales. However, for the WOMAC-F, a significantly greater difference in the dynamics of the indicators was found in the With Metf group compared to the Without Metf group (Table 2).

**TABLE 2:** Dynamics of WOMAC index indicators in the with and without Metf groups.

Indicator	Group	Before treatment	After 1 month	After 3 months	p-value
Pain Scale (P)	Without Metf (n = 26)	7 (5–8)	6 (6–7)	6 (5.25–7)	**p < 0.001** (F)
	With Metf (n = 34)	7 (6–8)	6 (5–7.75)	6 (5–7)	**p < 0.001** (F)
Stiffness Scale (S)	Without Metf (n = 26)	4 (3–4)	4 (3–4)	4 (3–4)	**p = 0.003** (F)
	With Metf (n = 34)	4 (3–4)	4 (3–4)	4 (3–4)	**p < 0.001** (F)
Function Scale (F)	Without Metf (n = 26)	18 (16–19.75)	18 (16–20)	16 (15–18.75)	**p < 0.001** (F)
	With Metf (n = 34)	19 (17–20)	17 (16–20)	17 (15–18)	**p < 0.001** (F)
Overall Score (OS)	Without Metf (n = 26)	28 (28–31.75)	27.5 (25–31)	26 (22.25–28)	**p < 0.001** (F)
	With Metf (n = 34)	29.5 (27–32.75)	28 (24–31)	26 (23.25–28.75)	**p < 0.001** (F)

Median and interquartile range (IQR) were used to summarize the data.

p - the statistical difference observed within a single group before treatment, after 1 month, and after 3 months [Friedman test (F)].

Statistically significant p-values are highlighted in bold.

In both studied groups, significant changes were observed in the Lequesne Algofunctional Index. In the Without Metf group, the analysis demonstrated significant changes in the LI-PA, LI-FIA, and LI-OS scales. Significant differences in the LI-FIA scale were observed between before treatment and after 1 month. Additionally, notable changes were identified in the LI-FIA and LI-OS scales between after 1 month and after 3 months (Table 3).

**TABLE 3:** Lequesne algofunctional index dynamics in the with and without Metf groups across treatment periods.

Indicator	Group	Before treatment	After 1 month	After 3 months	p-value
Pain Assessment (PA)	Without Metf (n = 26)	4 (4–4)	4 (4–4)	4 (3–4)	**p = 0.045** (F)
	With Metf (n = 34)	4 (4–5)	4 (3–4)	4 (3–4)	**p < 0.001** (F)
Functional Impairment Assessment (FIA)	Without Metf (n = 26)	4 (4–4)	4 (4–4)	4 (4–4)	**p = 0.006** (F)
	With Metf (n = 34)	4 (3–4)	3 (3–4)*	3 (3–3)***	**p < 0.001** (F)
Overall Score (OS)	Without Metf (n = 26)	8 (8–8)	8 (7–8.75)	7 (7–8)	**p < 0.001** (F)
	With Metf (n = 34)	8 (7–9)	7 (7–8)	7 (6–7)***	**p < 0.001** (F)

Median and interquartile range (IQR) were used to summarize the data.

p - the statistical difference observed within a single group before treatment, after 1 month, and after 3 months [Friedman test (F)].

* (p ≤ 0.05), ** (p ≤ 0.01), *** (p ≤ 0.001) – the statistical difference between the Without Metf and With Metf groups in a single observation period [Mann-Whitney U-test (MW)].

Statistically significant p-values are highlighted in bold.

In the With Metf group, significant changes were observed across all Lequesne Algofunctional Index scales during treatment. Notable differences were found in the LI-PA and LI-OS scales between before treatment and after 1 month, before treatment and after 3 months, and after 1 month and after 3 months. Significant changes were also observed in the LI-FIA scale between before treatment and after 3 months, and between after 1 month and after 3 months (Table 3).

A significantly greater difference in the dynamics of the LI-PA and LI-FIA scales was found in the With Metf group compared to the Without Metf group (Table 3).

Significant statistical differences between the groups were observed in the Lequesne Algofunctional Index scales, particularly in the LI-FIA and LI-OS, after 3 months (Table 3).

In the Without Metf group, the KOOS scales analysis revealed significant changes throughout treatment, particularly in the KOOS-FS/R and KOOS-KRQL scales. Significant changes were observed in the KOOS-FS/R scale and the KOOS-KRQL scale between before treatment and after 3 months, and after 1 month and after 3 months (Table 4).

**TABLE 4:** KOOS scale dynamics in the with and without Metf groups across treatment periods.

Indicator	Group	Before treatment	After 1 month	After 3 months	p-value
Pain (P)	Without Metf (n = 26)	66 (65–69.75)	67 (64–70)	67 (64–71)	p = 0.078 (F)
	With Metf (n = 34)	67.5 (63.25–70)	68 (64–70)	68.5 (65.5–72.75)	**p = 0.002** (F)
Other Symptoms (OS)	Without Metf (n = 26)	65 (62.25–68)	65 (63–69)	65 (63–69)	p = 0.228 (F)
	With Metf (n = 34)	66 (64–68.75)	66 (64–69.75)	68 (65–70.75)	**p < 0.001** (F)
Function in Daily Living (FDL)	Without Metf (n = 26)	71.5 (69.25–74)	72 (70–74)	72 (71–74)	p = 0.102 (F)
	With Metf (n = 34)	71 (68–74.75)	71 (69–75.75)	74.5 (70.25–78.5)	**p < 0.001** (F)
Function in Sport/Recreation (FS/R)	Without Metf (n = 26)	52 (49.25–54)	52 (49.25–55)	53.5 (50.5–55)	**p = 0.016** (F)
	With Metf (n = 34)	51 (48–53)	52 (48–54.75)	54 (50.25–56.5)	**p < 0.001** (F)
Knee-Related Quality of Life (KRQL)	Without Metf (n = 26)	59.5 (55.25–62)	59.5 (55.5–62)	60.5 (57.25–62.75)	**p < 0.001** (F)
	With Metf (n = 34)	59.5 (57–63)	60.5 (58–64.75)	63 (60–67)	**p < 0.001** (F)

Median and interquartile range (IQR) were used to summarize the data.

p - the statistical difference observed within a single group before treatment, after 1 month, and after 3 months (Friedman test (F)).

* (p ≤ 0.05), ** (p ≤ 0.01), *** (p ≤ 0.001) – the statistical difference between the Without Metf and With Metf groups in a single observation period (Mann-Whitney U-test (MW)).

Statistically significant p-values are highlighted in bold.

In the With Metf group, significant changes were observed across the KOOS scales – KOOS-P, KOOS-OS, KOOS-FDL, KOOS-FS/R, and KOOS- KRQL – during treatment. Notable changes were found between before treatment and after 1 month in the KOOS-OS and KOOS-FDL scales, and between before treatment and after 3 months in the KOOS-OS, KOOS-FDL, KOOS-FS/R, and KOOS- KRQL scales. Additionally, significant changes in the KOOS-P, KOOS-OS, and KOOS-FDL scales were observed between after 1 month and after 3 months (Table 4).

Additionally, a higher statistical significance of changes in the indicators was observed in the With Metf group for the KOOS-P, KOOS-OS, KOOS-FDL, and KOOS-FS/R scales, compared to the Without Metf group (Table 4).

In the Without Metf group, significant changes were noted in the VAS-P, VAS-FL, VAS-S, VAS-PA, and VAS-M scales throughout treatment. Notable differences were observed in the VAS-FL, VAS-S, and VAS-PA, and VAS-M scales between after 1 month and after 3 months, as well as in the VAS-PA and VAS-M scales between between before treatment and after 1 month (Table 5).

**TABLE 5:** Analysis of VAS changes in the with and without Metf groups across treatment periods.

Indicator	Group	Before treatment	After 1 month	After 3 months	p-value
Pain (P)	Without Metf (n = 26)	42.5 (37–46.25)	42.5 (39–45)	43.5 (40–47)	**p = 0.009** (F)
	With Metf (n = 34)	39 (36.25–43.5)	37 (34.25–40)**	35 (31–37.75)***	**p < 0.0010** (F)
Functional Limitations (FL)	Without Metf (n = 26)	37.5 (32–42.75)	38 (32.25–41)	36.5 (30–40)	**p = 0.008** (F)
	With Metf (n = 34)	34.5 (29–37)	32 (30–35)*	30 (27–35)**	**p < 0.001** (F)
Stiffness (S)	Without Metf (n = 26)	35.5 (34–41)	35 (32–40)	35 (30–39.5)	**p = 0.01** (F)
	With Metf (n = 34)	36.5 (31.25–40.75)	32.5 (30–36.5)	31 (27.75–35)	**p < 0.001** (F)
Physical Activity and Mobility (PAM)	Without Metf (n = 26)	35.5 (29–38.75)	32.5 (25.5–37)	32.5 (25–35)	**p < 0.001** (F)
	With Metf (n = 34)	36 (30.25–40)	33.5 (30–40)	31 (25.5–37)	**p < 0.001** (F)

Median and interquartile range (IQR) were used to summarize the data.

p - the statistical difference observed within a single group before treatment, after 1 month, and after 3 months [Friedman test (F)].

* (p ≤ 0.05), ** (p ≤ 0.01), *** (p ≤ 0.001) – the statistical difference between the Without Metf and With Metf groups in a single observation period [Mann-Whitney U-test (MW)].

Statistically significant p-values are highlighted in bold.

In the With Metf group, significant changes were observed throughout treatment in the VAS-P, VAS-FL, VAS-S, VAS-PA, and VAS-M scales. Statistically significant differences were found between before treatment and after 1 month, before treatment and after 3 months, as well as between after 1 month and after 3 months for all these scales (Table 5).

Furthermore, a greater statistical significance in the changes of the indicators was found in the With Metf group for your VAS-P, VAS-FL, and VAS-S scales, compared to the Without Metf group (Table 5).

Statistical differences between the groups were observed in the VAS-P and VAS-FL scales after 1 and 3 months. Furthermore, a significant difference was noted in the VAS-S scale between the groups after 3 months (Table 5).

In the Without Metf group, significant changes were observed in the TUG and 6MWT scales throughout the treatment period. Statistically significant differences were found between before treatment and after 3 months, as well as between after 1 month and after 3 months for both scales. Additionally, changes in the TUG scale were significant between before treatment and after 1 month (Table 6).

**TABLE 6:** Changes in TUG and 6MWT scales across treatment periods in the with and without Metf groups.

Indicator	Group	Before treatment	After 1 month	After 3 months	p-value
Timed Up and Go (TUG)	Without Metf (n = 26)	15.5 (13–19)	14.5 (12–16)	13 (12–15)	**p < 0.001** (F)
	With Metf (n = 34)	14 (12–17)	12 (10–15)*	12 (10–15)	**p < 0.001** (F)
6-Minute Walk Test (6MWT)	Without Metf (n = 26)	345 (335.25–353.75)	345 (335.25–356)	350 (340–358.75)	**p < 0.001** (F)
	With Metf (n = 34)	349.5 (335.25–359.75)	355 (340.5–365)*	355 (346.25–366)*	**p < 0.001** (F)

Median and interquartile range (IQR) were used to summarize the data.

p - the statistical difference observed within a single group before treatment, after 1 month, and after 3 months [Friedman test (F)].

* (p ≤ 0.05), ** (p ≤ 0.01), *** (p ≤ 0.001) – the statistical difference between the Without Metf and With Metf groups in a single observation period [Mann-Whitney U-test (MW)].

Statistically significant p-values are highlighted in bold.

In the With Metf group, significant changes were observed in the TUG and 6MWT scales throughout the treatment period. Statistically significant differences were found in all indicators of both scales between before treatment and after 1 month, before treatment and after 3 months, as well as between after 1 month and after 3 months (Table 6).

Between the groups, statistical differences were observed for the TUG and 6MWT scales after 1 month, with a further significant difference noted in the 6MWT scale after 3 months (Table 6).

**FIGURE 1:**
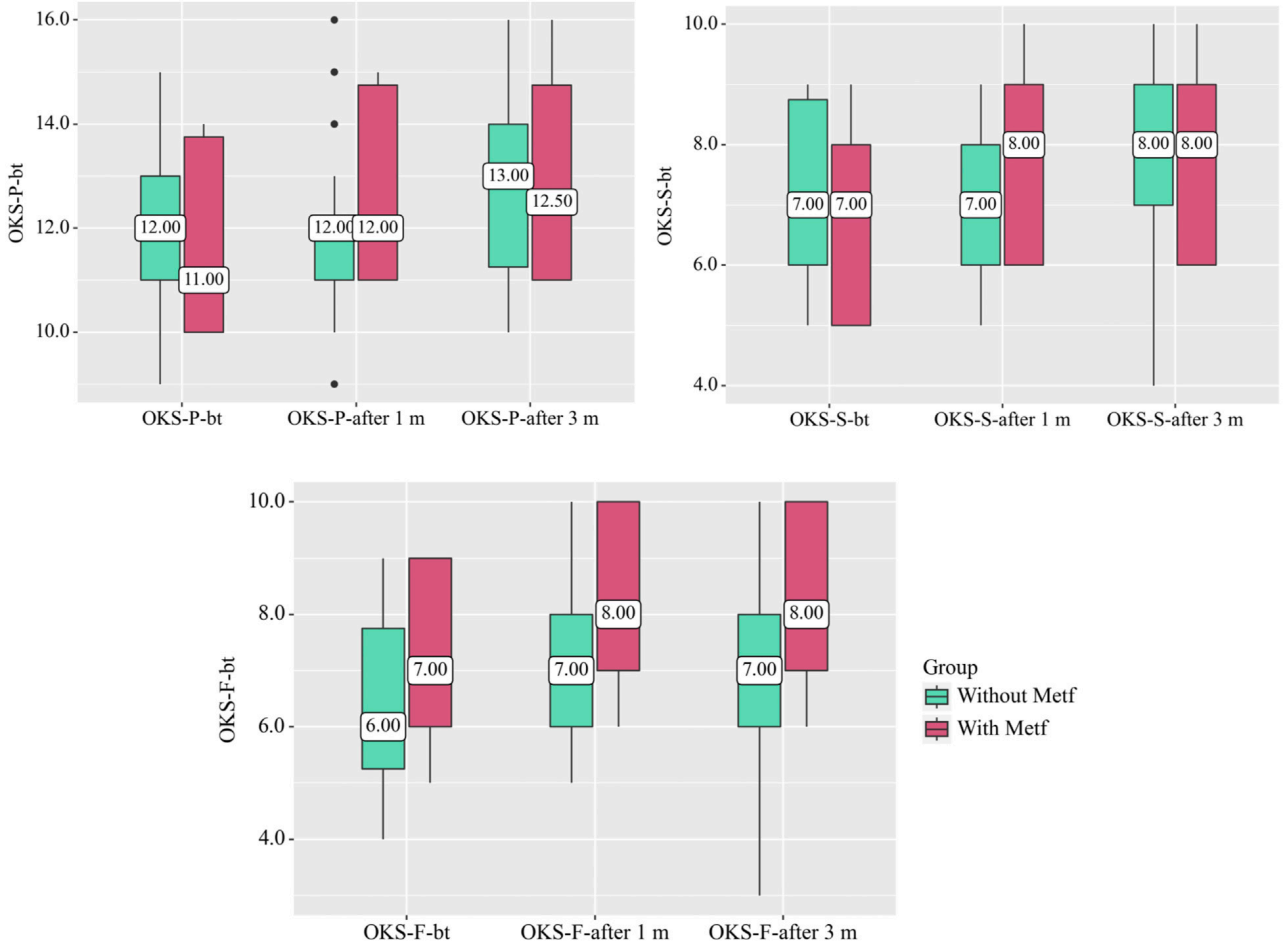
Changes in OKS Questionnaire Scales in the With and Without Metf Groups Across Treatment Periods. Statistical analysis was performed using the Friedman test for differences within groups over time, with *post hoc* comparisons conducted using the Conover-Iman test with Holm correction. Differences between groups at each time point were assessed using the Mann-Whitney U-test.

In the Without Metf group, statistically significant changes were observed across the OKS questionnaire scales during treatment, including OKS-P (p = 0.005) (Friedman test), OKS-S (p = 0.002) (Friedman test), and OKS-F (p = 0.001) (Friedman test). Additionally, significant differences were found in the OKS-F scale before treatment and after 1 month (p < 0.001) (Conover-Iman test with Holm correction), in the OKS-S scale before treatment and after 3 months, and in the OKS-F scale between after 1 month and after 3 months (p = 0.008) (Conover-Iman test with Holm correction) (Figure 1).

In the With Metf group, statistically significant changes were observed across all OKS questionnaire scales throughout treatment (p < 0.001) (Friedman test). Significant differences were also found between before treatment and after 1 month for all scales of the questionnaire (p < 0.001) (Conover-Iman test with Holm correction). Additionally, statistically significant changes were noted between after 1 month and after 3 months for all OKS scales (p < 0.001) (Conover-Iman test with Holm correction) (Figure 1).

Statistically significant differences between the groups were also observed in the OKS-F scale after 1 month (p = 0.043) (Mann-Whitney U-test) and after 3 months (p = 0.032) (Mann-Whitney U-test) (Figure 1).

**FIGURE 2:**
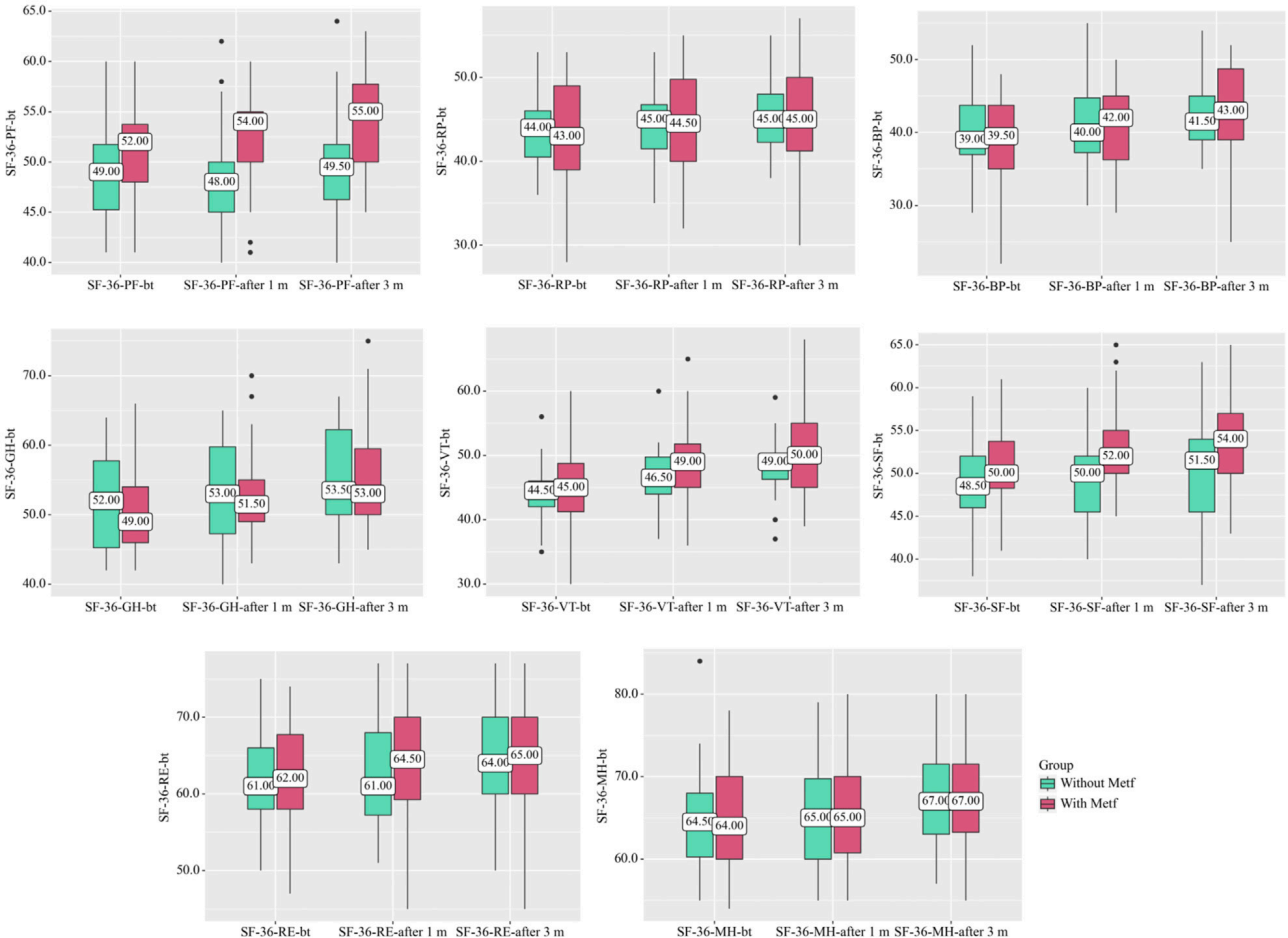
SF-36 Scale Dynamics in the With and Without Metf Groups Across Treatment Periods. Statistical analysis was performed using the Friedman test for differences within groups over time, with post hoc comparisons conducted using the Conover-Iman test with Holm correction. Differences between groups at each time point were assessed using the Mann-Whitney U-test.

In the Without Metf group, statistically significant changes were observed across several SF-36 scales throughout treatment. Specifically, SF-36-RP (p = 0.004) (Friedman test) [before treatment vs. after 1 month: p = 0.035, before treatment vs. after 3 months: p < 0.001, after 1 month vs. after 3 months: p = 0.035 (Conover-Iman test with Holm correction)]. SF-36-GH (p < 0.001) (Friedman test) [before treatment vs. after 3 months: p < 0.001, after 1 month vs. after 3 months: p < 0.001 (Conover-Iman test with Holm correction)], SF-36-VT (p < 0.001) (Friedman test) [before treatment vs. after 1 month: p < 0.001, before treatment vs. after 3 months: p < 0.001, after 1 month vs. after 3 months: p = 0.014 (Conover-Iman test with Holm correction)], SF-36-SF (p = 0.015) (Friedman test) [before treatment vs. after 3 months: p = 0.032, after 1 month vs. after 3 months: p = 0.046 (Conover-Iman test with Holm correction)], SF-36-RE showed (p < 0.001) (Friedman test) [before treatment vs. after 3 months: p < 0.001, after 1 month vs. after 3 months: p = 0.001 (Conover-Iman test with Holm correction)], and SF-36-MH (p < 0.001) (Friedman test) [before treatment vs. after 3 months: p < 0.001, after 1 month vs. after 3 months: p < 0.001 (Conover-Iman test with Holm correction)] (Figure 2).

In the With Metf group, statistically significant changes were observed across several SF-36 scales throughout treatment. Specifically, SF-36-PF (p < 0.001) (Friedman test) [before treatment vs. after 1 month: p = 0.004, before treatment vs. after 3 months: p < 0.001, after 1 month vs. after 3 months: p = 0.039 (Conover-Iman test with Holm correction)], SF-36-BP (p < 0.001) (Friedman test) [before treatment vs. after 1 month: p < 0.001, before treatment vs. after 3 months: p < 0.001, after 1 month vs. after 3 months: p = 0.004 (Conover-Iman test with Holm correction)], SF-36-GH (p < 0.001) (Friedman test) [before treatment vs. after 1 month: p < 0.001, before treatment vs. after 3 months: p < 0.001, after 1 month vs. after 3 months: p = 0.007 (Conover-Iman test with Holm correction)], SF-36-VT (p < 0.001) (Friedman test) [before treatment vs. after 1 month: p < 0.001, before treatment vs. after 3 months: p < 0.001, after 1 month vs. after 3 months: p = 0.012 (Conover-Iman test with Holm correction)], SF-36-RE (p < 0.001) (Friedman test) [before treatment vs. after 1 month: p = 0.004, before treatment vs. after 3 months: p < 0.001, after 1 month vs. after 3 months: p = 0.045 (Conover-Iman test with Holm correction)], and SF-36-MH (p < 0.001) (Friedman test) [before treatment vs. after 1 month: p = 0.003, before treatment vs. after 3 months: p < 0.001, after 1 month vs. after 3 months: p = 0.023 (Conover-Iman test with Holm correction)] (Figure 2).

A statistically significant difference was also found in the indicators between the groups on the scales SF-36-PF after 1 month (р = 0.002) (Mann-Whitney U-test) and SF-36-SF after 1 month (р = 0.005) (Mann-Whitney U-test), after 3 months (р = 0.020) (Mann-Whitney U-test) (Figure 2).

In the Without Metf group, significant changes in BMI and WHR were observed over the course of treatment. Furthermore, statistically significant differences in BMI and WHR were identified between before treatment and after 1 month of treatment. Similarly, significant changes in BMI and WHR were observed between after 1 month and after 3 months of treatment (Table 7).

**TABLE 7:** Changes in BMI, WHR, and WHtR across treatment periods in the with and without Metf groups.

Indicator	Group	Before treatment	After 1 month	After 3 months	p-value
Body Mass Index, kg/m^2^ (BMI)	Without Metf (n = 26)	29.87 (27.35–31.26)	29.61 (27.15–31.6)	28.99 (26.57–30.51)	**p = 0.019** (F)
	With Metf (n = 34)	28.02 (26.39–30.19)	26.52 (25.26–29.32)*	24.98 (24.68–27.54)**	**p < 0.001** (F)
Waist-to-Hip Ratio, (WHR)	Without Metf (n = 26)	0.96 (0.92–0.99)	0.94 (0.91–0.96)	0.94 (0.88–0.96)	**p < 0.001** (F)
	With Metf (n = 34)	0.99 (0.94–1.03)	0.91 (0.85–0.96)	0.81 (0.75–0.86)***	**p < 0.001** (F)
Waist-to-Height Ratio, (WHtR)	Without Metf (n = 26)	0.56 (0.51–0.6)	0.55 (0.52–0.59)	0.55 (0.51–0.59)	p = 0.762 (F)
	With Metf (n = 34)	0.54 (0.48–0.64)	0.5 (0.45–0.56)*	0.5 (0.45–0.52)**	**p < 0.001** (F)

Median and interquartile range (IQR) were used to summarize the data.

p - the statistical difference observed within a single group before treatment, after 1 month, and after 3 months [Friedman test (F)].

* (p ≤ 0.05), ** (p ≤ 0.01), *** (p ≤ 0.001) – the statistical difference between the Without Metf and With Metf groups in a single observation period [Mann-Whitney U-test (MW)].

Statistically significant p-values are highlighted in bold.

In the With Metf group, significant changes were observed in BMI, WHR, and WHtR values throughout the course of treatment. Furthermore, statistically significant differences were identified for all indicators between before treatment and after 1 month, before treatment and after 3 months, as well as between after 1 month and after 3 months within this group (Table 7).

Moreover, a more significant statistical difference in the changes of the indicators was observed in the With Metf group for the BMI and WHtR scales, compared to the Without Metf group (Table 7).

Between the groups, significant statistical differences were observed after 1 month for BMI and WHtR, and after 3 months for BMI, WHR, and WHtR (Table 7).

In the Without Metf group, significant statistical changes were observed in FPG level throughout the course of treatment. Furthermore, notable differences in FPG were found between the measurements taken at 1 month and 3 months (Table 8).

**TABLE 8:** Changes in glucose tolerance indicators during treatment.

Indicator	Group	Before treatment	After 1 month	After 3 months	p-value
Fasting plasma glucose, mmol/L (FPG)	Without Metf (n = 26)	6.44 (6.32–6.54)	6.38 (6.24–6.51)	6.4 (6.25–6.41)	**p = 0.006** (F)
	With Metf (n = 34)	6.38 (6.18–6.45)	6.01 (5.85–6.04)***	5.53 (5.49–5.7)***	**p < 0.001** (F)
Glycated hemoglobin, % (HbA1c)	Without Metf (n = 26)	6 (5.9–6.1)	6 (6–6.1)	6 (5.9–6.1)	P = 0.162 (F)
	With Metf (n = 34)	6 (5.9–6.1)	5.8 (5.7–6)***	5.65 (5.5–5.7)***	**p < 0.001** (F)
HOMA-IR	Without Metf (n = 26)	2.74 (2.68–2.83)	2.75 (2.7–2.85)	2.79 (2.67–2.86)	P = 0.542 (F)
	With Metf (n = 34)	2.69 (2.62–2.8)	2.62 (2.52–2.71)**	2.49 (2.4-2.59)***	**p < 0.001** (F)
C-peptide, ng/mL	Without Metf (n = 26)	3.08 (2.96–3.2)	3 (2.9–3.09)	3.02 (2.93–3.18)	P^a^ = 0.211 (F)
	With Metf (n = 34)	3.02 (2.94–3.12)	2.94 (2.81–3.01)	2.84 (2.75–2.93)***	**p < 0.001** (F)

Median and interquartile range (IQR) were used to summarize the data.

p - the statistical difference observed within a single group before treatment, after 1 month, and after 3 months [Friedman test (F)].

* (p ≤ 0.05), ** (p ≤ 0.01), *** (p ≤ 0.001) – the statistical difference between the Without Metf and With Metf groups in a single observation period [Mann-Whitney U-test (MW)].

Statistically significant p-values are highlighted in bold.

In the With Metf group, statistically significant changes were observed throughout the treatment in the levels of FPG, HbA1c, HOMA-IR, and C-peptide. Additionally, significant changes were found across all indicators when comparing measurements before treatment to after 1 month, before treatment to after 3 months, and between after 1 month and after 3 months (Table 8).

Additionally, the With Metf group showed a more pronounced statistical difference in the changes of the FPG, HbA1c, HOMA-IR, and C-peptide indicators compared to the Without Metf group (Table 8).

Statistically significant differences between the groups were observed after 1 month for all indicators, with the exception of C-peptide. After 3 months, significant differences were found across all studied parameters (Table 8).

In the Without Metf group, statistically significant changes were observed throughout the treatment in the levels of NLR, CRP, MA, and SOD. Additionally, significant changes in NLR and SOD were found between before treatment and after 1 month. Statistically significant changes in NLR and CRP were observed between before treatment and after 3 months. Furthermore, significant differences were noted in NLR, CRP, MA, and SOD between after 1 month and after 3 months (Table 9).

**TABLE 9:** Changes in inflammatory markers and antioxidant Enzyme levels throughout the treatment period.

Indicator	Group	Before treatment	After 1 month	After 3 months	p-value
Neutrophil-to-Lymphocyte Ratio (NLR)	Without Metf (n = 26)	2.48 (2.3–2.62)	2.46 (2.29–2.62)	2.33 (2.21–2.54)	**p < 0.001** (F)
	With Metf (n = 34)	2.47 (2.33–2.71)	2.35 (2.19–2.61)	2.29 (2.15–2.56)	**p < 0.001** (F)
C-Reactive Protein, mg/L (CRP)	Without Metf (n = 26)	4.5 (3.79–5.09)	4.46 (3.71–5.04)	4.35 (3.63–4.91)	**p < 0.001** (F)
	With Metf (n = 34)	4.56 (4.23–5.2)	4.5 (4.19–5.04)	4.2 (4.04–4.57)	**p < 0.001** (F)
Hydroxyproline, mg/L (HP)	Without Metf (n = 26)	7.71 (6.74–8.38)	7.77 (6.56–8.36)	7.71 (6.55–7.96)	p = 0.240 (F)
	With Metf (n = 34)	7.04 (6.33–7.85)	6.9 (6.29–7.66)*	6.5 (5.9–7.39)*	**p < 0.001** (F)
Malondialdehyde, µmol/L (MA)	Without Metf (n = 26)	7.09 (4.84–8.29)	6.95 (4.73–8.19)	6.86 (4.65–7.81)	**p = 0.012** (F)
	With Metf (n = 34)	6.73 (5.57–8.03)	6 (5.02–7.05)	5.32 (4.63–6.58)	**p < 0.001** (F)
Superoxide Dismutase, U/mL (SOD)	Without Metf (n = 26)	195.02 (189.05–204.3)	197.03 (190.77–208.99)	206 (196.04–209.25)	**p < 0.001** (F)
	With Metf (n = 34)	195.44 (189–210.4)	202.52 (193.98–214.46)	206.87 (201.04–217)	**p < 0.001** (F)
α_1_-Antitrypsin, g/L (α_1_-AT)	Without Metf (n = 26)	1.6 (1.52–1.65)	1.61 (1.52–1.65)	1.6 (1.55–1.65)	p = 0.228 (F)
	With Metf (n = 34)	1.65 (1.52–1.70)	1.64 (1.55–1.7)	1.65 (1.58–1.7)	**p = 0.013** (F)

Median and interquartile range (IQR) were used to summarize the data.

p - the statistical difference observed within a single group before treatment, after 1 month, and after 3 months [Friedman test (F)].

* (p ≤ 0.05), ** (p ≤ 0.01), *** (p ≤ 0.001) – the statistical difference between the Without Metf and With Metf groups in a single observation period [Mann-Whitney U-test (MW)].

Statistically significant p-values are highlighted in bold.

In the With Metf group, statistically significant changes were observed during the treatment period in the levels of NLR, CRP, HP, MA, SOD, and α1-AT. Significant changes in NLR, CRP, MA, and SOD were found between before treatment and after 1 month. Additionally, statistically significant changes in NLR, CRP, HP, MA, and SOD were observed between before treatment and after 3 months. Furthermore, significant differences in NLR, CRP, HP, MA, SOD, and α_1_-AT were noted between after 1 month and after 3 months (Table 9).

Furthermore, a more significant statistical difference in the changes of the HP, MA, and α_1_-AT indicators was observed in the With Metf group compared to the Without Metf group (Table 9).

Statistically significant differences between the groups were observed in HP levels after 1 month and after 3 months (Table 9).

In the Without Metf group, statistically significant changes were observed in LDL levels throughout the treatment period. Additionally, significant changes in LDL levels were found between before treatment and after 3 months, as well as between after 1 month and after 3 months (Table 10).

**TABLE 10:** Changes in lipid profile parameters throughout the treatment period.

Indicator	Group	Before treatment	After 1 month	After 3 months	p-value
Total Cholesterol, mmol/L (TC)	Without Metf (n = 26)	5.6 (5.39–5.88)	5.59 (5.36–5.91)	5.58 (5.37–5.77)	P = 0.205 (F)
	With Metf (n = 34)	5.43 (5.32–5.61)	5.34 (5.23–5.53)**	5.27 (5.12–5.48)***	**p < 0.001** (F)
Low-Density Lipoprotein, mmol/L (LDL)	Without Metf (n = 26)	3.5 (3.19–3.68)	3.49 (3.19–3.76)	3.41 (3.2–3.67)	**p = 0.006** (F)
	With Metf	3.37 (2.98–3.51)	3.15 (2.95–3.39)	3.15 (2.95–3.40)**	**p < 0.001** (F)
High-Density Lipoprotein, mmol/L (HDL)	Without Metf (n = 26)	1.23 (1.1–1.4)	1.23 (1.08–1.39)	1.27 (1.13–1.39)	p = 0.218 (F)
	With Metf (n = 34)	1.19 (1.12–1.25)	1.27 (1.18–1.33)	1.31 (1.22–1.40)	**p < 0.001** (F)
Triglycerides, mmol/L (TG)	Without Metf (n = 26)	1.94 (1.86–2.02)	1.98 (1.83–2.05)	1.88 (1.78–2)	p = 0.575 (F)
	With Metf (n = 34)	2 (1.85–2.11)	1.97 (1.83–2.08)	1.98 (1.82–2.08)	**p < 0.001** (F)

Median and interquartile range (IQR) were used to summarize the data.

p - the statistical difference observed within a single group before treatment, after 1 month, and after 3 months [Friedman test (F)].

* (p ≤ 0.05), ** (p ≤ 0.01), *** (p ≤ 0.001) – the statistical difference between the Without Metf and With Metf groups in a single observation period [Mann-Whitney U-test (MW)].

Statistically significant p-values are highlighted in bold.

In the Metf group, statistically significant changes were observed throughout the treatment period in TC, LDL, HDL, and TG levels. Additionally, significant changes were found in TC and LDL levels between before treatment and after 1 month. TC and HDL levels exhibited statistically significant changes between before treatment and after 3 months. Furthermore, TC, LDL, HDL, and TG levels showed significant changes between after 1 month and after 3 months (Table 10).

In addition, a more notable statistical difference in the changes of the TC, LDL, HDL, and TG indicators was found in the With Metf group compared to the Without Metf group (Table 10).

Statistically significant differences between the groups were found in TC and LDL levels after 1 and 3 months (Table 10).

### 3.2 Assessment of osteoarthritis exacerbation parameters

Over a 3-month period, various aspects of osteoarthritis (OA) exacerbations were investigated, including the duration of flare-ups (OA exc. dur., ≤7 days or >7 days), their frequency (OA exc. freq., ≤2 times or >2 times), and the need for additional medical interventions such as injections or physiotherapy (OA exc. med. int.). Other factors assessed included the requirement for work leave or exemption due to exacerbations (OA exc. work leave), the occurrence of accompanying symptoms (OA exc. acc. sym., e.g., swelling, redness), and the necessity to adjust NSAID therapy by increasing either the dose (NSAIDs dose) or duration of use (NSAID dur.). These parameters provided insight into the severity and impact of OA exacerbations on patient management (Table 11).

**TABLE 11:** Indicators of osteoarthritis exacerbation characteristics based on group.

Variable	Categories	Group		p
		Without Metf (n = 26)	With Metf (n = 34)	
OA exc. dur	≤7 days	11 (42.3)	14 (41.2)	0.952
	>7 days	15 (57.7)	20 (58.8)	
OA exc. freq	≤2 times	13 (50.0)	16 (47.1)	0.834
	>2 times	13 (50.0)	18 (52.9)	
OA exc. med. int	no	15 (57.7)	24 (70.6)	0.414
	yes	11 (42.3)	10 (29.4)	
OA exc. work leave	no	19 (73.1)	27 (79.4)	0.759
	yes	7 (26.9)	7 (20.6)	
OA exc. acc. sym	no	20 (76.9)	29 (85.3)	0.507
	yes	6 (23.1)	5 (14.7)	
NSAIDs dose	no	18 (69.2)	27 (79.4)	0.386
	yes	8 (30.8)	7 (20.6)	
NSAID dur	no	16 (61.5)	27 (79.4)	0.156
	yes	10 (38.5)	7 (20.6)	

The number of patients (percentage) p–Fisher’s exact test.

No statistically significant difference was found between these indicators in the studied groups during the treatment period. Additionally, an analysis of the interrelationships among the investigated indicators of Osteoarthritis Exacerbation Characteristics was conducted, and a prognostic model was developed to predict the need for increased NSAID doses in patients with osteoarthritis and impaired glucose tolerance, based on factors such as VAS-P-after 3 months, OKS-F-after 3 months, HbA1c-after 3 months, C-peptide-after 3 months, CRP-after 3 months, HP-after 3 months, and α1-AT-after 3 months using binary logistic regression. The model was constructed using 60 observations, and the relationship between these variables is described by the following equation:

$$P=1\;/\;\left(1+e^{‐z}\right)\times 100\%$$

$$z=‐10,247+0,346X_{\text{VAS}‐P‐\text{after}\;3\;m}+1,011X_{\text{OKS}‐F‐\text{after}\;3\;m}\;‐\;13,099X_{\text{HbA}1c‐\text{after}\;3\;m}+11,210X_{C‐\text{peptide}‐\text{after}\;3\;m}\;‐\;3,489X_{\text{CRP}‐\text{after}\;3\;m}+1,807X_{\text{HP}‐\text{after}\;3\;m}+19,109X_{\alpha 1‐\text{AT}‐\text{after}\;3\;m}$$

where P represents the probability estimate for “yes,” z denotes the value of the logistic function, X_VAS-P-after 3 m_ refers to VAS-P-after 3 m, X_OKS-F-after_ 3 m refers to OKS-F-after 3 m, X_hba1c-after 3 m_ refers to HbA1c-after 3 m, X_C-peptide-after 3 m_ refers to C-peptide-after 3 m, X_CRP-after 3 m_ refers to CRP-after 3 m, XH_P-after 3 m refers_ to HP-after 3 m, and X_α1-at-after 3 m_ refers to α_1_-AT-after 3 months.

The resulting regression model, in terms of the alignment between the predicted and observed values upon the inclusion of predictors compared to the model without predictors, is statistically significant (p < 0.001). The Nagelkerke pseudo-R^2^ was 67.9% (Table 12).

**TABLE 12:** Characteristics of the association between predictors of the model and the odds of the need for increased NSAID doses.

Predictors	Unadjusted		Adjusted	
	COR; 95% CI	p	AOR; 95% CI	p
VAS-P-after 3 m	1.079; 1.005–1.158	0.037*	1.414; 1.119–1.788	0.004*
OKS-F-after 3 m	1.308; 0.894–1.914	0.167	2.748; 1.105–6.828	0.030*
HbA1c-after 3 m	1.867; 0.137–25.508	0.640	0.000; 0.000–0.075	0.015*
C-peptideafter 3 m	74.040; 2.754–1990.219	0.010*	73,887.578; 5.382–1,014,843,245.924	0.021*
CRP-after 3 m	0.603; 0.253–1.436	0.253	0.031; 0.003–0.347	0.005*
HP-after 3 m	1.975; 1.139–3.425	0.015*	6.095; 1.626–22.851	0.007*
α_1_-AT-after 3 m	0.058; 0.000–36.017	0.385	198,996,938.868; 8.406–4,712,523,809,095,451.000	0.027*

* – the effect of the predictor is statistically significant (p < 0.05).

**FIGURE 3:**
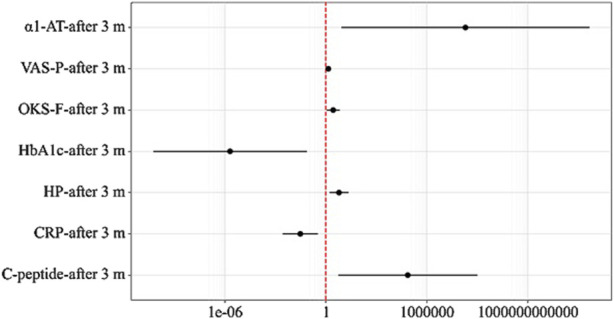
Odds ratios with 95% CI estimates for the studied predictors of the Need for Increased NSAID Doses.

An increase of 1 in VAS-P-after 3 months increased the odds of the need for increased NSAID doses by a factor of 1.414. An increase of 1 in OKS-F-after 3 months increased the odds of the need for increased NSAID doses by a factor of 2.748. An increase of 1 in HbA1c-after 3 months decreased the odds of the need for increased NSAID doses by a factor of 488,307.202. An increase of 1 in C-peptide-after 3 months increased the odds of the need for increased NSAID doses by a factor of 73,887.578. An increase of 1 in CRP-after 3 months decreased the odds of the need for increased NSAID doses by a factor of 32.756. An increase of 1 in HP-after 3 months increased the odds of the need for increased NSAID doses by a factor of 6.095. An increase of 1 in α1-AT-after 3 months increased the odds of the need for increased NSAID doses by a factor of 198,996,938.868 (Figure 3).

**FIGURE 4:**
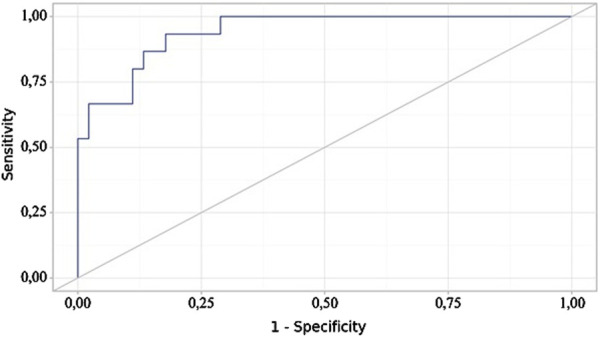
ROC curve illustrating the discriminatory ability of the regression model in predicting NSAIDs dose.

**FIGURE 5:**
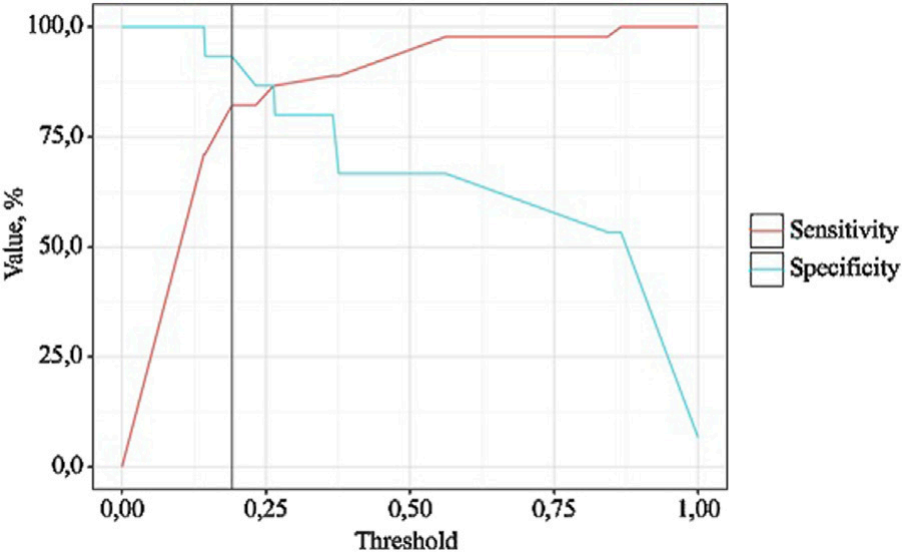
Analysis of model sensitivity and specificity depending on the threshold values of NSAIDs dose probability estimates.

The following curve was obtained when assessing the discriminatory ability of the regression model using ROC analysis (Figures 4, 5).

The probability estimate P is a statistically significant predictor of the Need for Increased NSAID Doses (AUC = 0.942; 95% CI: 0.858–1.000, p < 0.001). The threshold value of the probability estimate P at the cut-off point, corresponding to the highest Youden’s index, was 0.191. A “yes” was predicted when the probability estimate P was greater than or equal to this value. The sensitivity and specificity of the resulting predictive model were 93.3% and 82.2%, respectively.

A statistical analysis was conducted to evaluate the relationship between BMI and the duration of osteoarthritis exacerbations (OA exc. dur.) (Table 13).

**TABLE 13:** Analysis of BMI depending on OA exacerbation duration.

Indicator	Categories	BMI-after 1 m			p
		Me	Q_1_ – Q_3_	n	
OA exc. dur	≤7 days	27.01	24.84–28.16	25	0.015*
	>7 days	29.54	25.81–31.28	35	

* – the effect of the predictor is statistically significant (p < 0.05).

**FIGURE 6:**
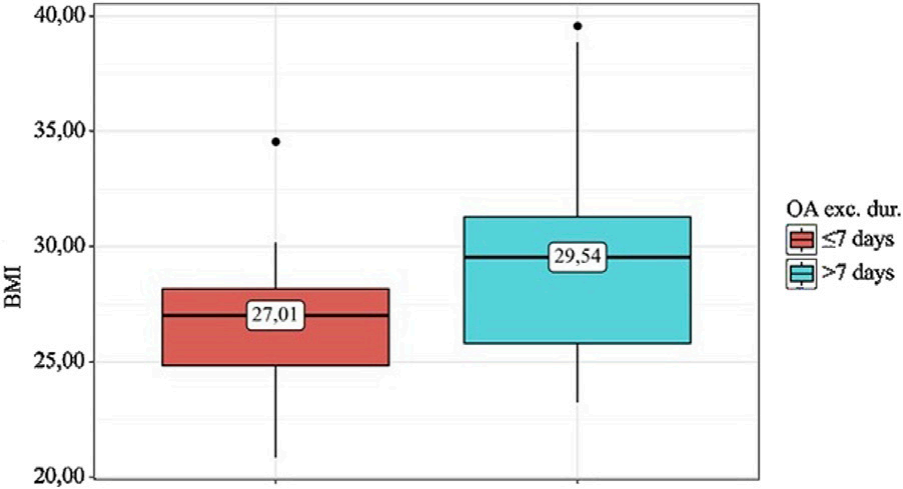
Analysis of BMI depending on OA exc. dur.

According to the presented table, when analyzing BMI depending on OA exc. dur., statistically significant differences were found (p = 0.015) (Figure 6).

**FIGURE 7:**
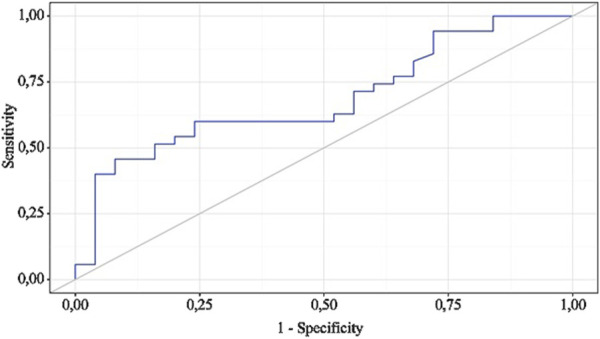
ROC curve characterizing the discriminatory ability of BMI-after 1 m in predicting OA exc. dur.

**FIGURE 8:**
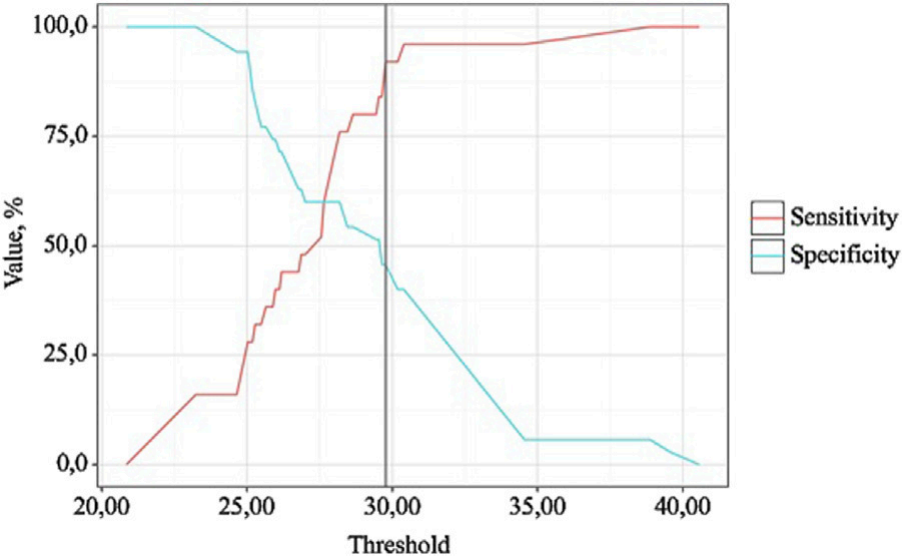
Analysis of the sensitivity and specificity of the model depending on the threshold values of the probability assessments for OA exc. dur.

The discriminatory capacity of >7 days from BMI was evaluated using ROC analysis, which yielded the following curve (Figures 7, 8).

BMI is a statistically significant predictor of OA exc. dur (AUC = 0.686; 95% CI: 0.553–0.820, p = 0.015). The threshold value of BMI at the cut-off point, corresponding to the highest Youden index, was 29.770. >7 days was predicted when BMI was equal to or greater than this value. The sensitivity and specificity of the obtained predictive model were 45.7% and 92.0%, respectively.

## 4 Discussion

The application of metformin in OA has garnered attention for its metabolic and anti-inflammatory properties, with additional implications for oxidative stress and lipid profile modulation (Song et al., 2022; Lai et al., 2022; Chen et al., 2022; Ragab et al., 2024). Previous studies have demonstrated that metformin exerts a beneficial effect on oxidative stress, an important factor in OA pathogenesis (Alimoradi et al., 2025; Zuliani et al., 2020; Arinno et al., 2023). Oxidative stress plays a significant role in the degeneration of cartilage and the progression of OA by promoting inflammation and joint tissue damage (Ansari et al., 2020; Liu et al., 2022). Our findings support this, as metformin treatment resulted in reduced levels of markers such as superoxide dismutase, which is associated with oxidative stress. This is consistent with previous research indicating that metformin can reduce oxidative stress and protect against joint degeneration in OA patients, potentially contributing to a slower progression of the disease (Xu et al., 2024; Ruan et al., 2022; Barnett et al., 2017; Hyun et al., 2013). Metformin exerts its therapeutic effects in OA through a combination of systemic and local mechanisms that address both metabolic and inflammatory pathways contributing to disease progression (Yao et al., 2023; Song et al., 2021; Wang et al., 2019). At the systemic level, metformin significantly reduces insulin resistance and hyperglycemia, which are strongly linked to the chronic low-grade inflammation characteristic of metabolic disorders, including IGT (Tsalamandris et al., 2019; Tizazu et al., 2019). By activating AMP-activated protein kinase (AMPK), metformin plays a pivotal role in inhibiting the mechanistic target of rapamycin (mTOR) signaling pathway (Amin et al., 2019; Nair et al., 2014; Putilin et al., 2020). This inhibition is critical, as mTOR activation is associated with chondrocyte hypertrophy, extracellular matrix breakdown, and cartilage degeneration, all of which are hallmark features of OA pathology (Fazio et al., 2024; Chawla et al., 2022; Dong and Jin, 2025).

Locally, metformin demonstrates potent anti-inflammatory properties by downregulating the production of pro-inflammatory cytokines such as interleukin-1β (IL-1β) and tumor necrosis factor-α (TNF-α) (Jia et al., 2024; Scafidi et al., 2024; Tsuji et al., 2020). These cytokines are major drivers of synovial inflammation, joint swelling, and cartilage erosion (Lubberts and van den Berg, 2003; Miller et al., 2014; Lubberts et al., 2001; Sokolove and Lepus, 2013).

Metformin has also demonstrated antiviral properties, particularly in inhibiting the replication of several viruses, including COVID-19 (Halabitska et al., 2024e; Petakh et al., 2022; Buchynskyi et al., 2023). Its potential to modulate viral infections, coupled with its anti-inflammatory effects, suggests that metformin may offer therapeutic benefits beyond metabolic conditions (Petakh et al., 2023a; Martin et al., 2023; Amengual-Cladera et al., 2024). Metformin exhibits antimicrobial properties, influencing pathogens and gut microbiota (Nosulenko et al., 2014; Jauvain et al., 2021; Bilyi et al., 2015; Garg and Mohajeri, 2024). Metformin affects stress responses, hormonal balance, and gut microbiota, potentially reducing inflammation and improving metabolic resilience (Topol and Kamyshny, 2013; Bilous et al., 2021; Petakh et al., 2023b).

Additionally, metformin’s effects on lipid profile are a notable area of interest (Gillani et al., 2021; Machado et al., 2012; Garimella et al., 2016). OA patients often present with metabolic disturbances, including dyslipidemia, which can exacerbate joint inflammation and cartilage degradation (Adam et al., 2024; Wei et al., 2023; Zhu et al., 2022). In our study, metformin led to significant improvements in lipid markers, including total cholesterol, LDL, HDL, and triglycerides. This is in line with other studies that have shown metformin’s ability to improve lipid profiles, suggesting that it may not only mitigate inflammation and oxidative stress but also correct underlying metabolic dysfunctions that worsen OA symptoms (Xing et al., 2022; Pradas et al., 2019; Zou et al., 2024). In fact, metformin’s ability to improve lipid metabolism may offer an additional mechanism for its positive effects in OA, as dyslipidemia is associated with increased risk of systemic inflammation and accelerated joint damage (Chen et al., 2022; Sobieh et al., 2023; Gkretsi et al., 2010; Mocanu et al., 2024).

When comparing our findings to other studies, the reduction in inflammatory markers such as C-reactive protein (CRP) and neutrophil-to-lymphocyte ratio with metformin use is also well-documented in the literature (Cameron et al., 2016; Hambly et al., 2023; Pitsavos et al., 2007; Rahnavard et al., 2022). These results highlight metformin’s dual role in both controlling blood glucose and exerting anti-inflammatory effects, which have been linked to improved clinical outcomes in OA patients (Veronese et al., 2019; Kim et al., 2022; Lin et al., 2023). While our study demonstrates significant improvements in pain, stiffness, and functional limitations, differences in patient populations, dosages, or treatment durations align with the variability observed in the broader literature (Magni et al., 2021; Ferreira et al., 2024; Nahin et al., 2016). These discrepancies emphasize the need for further research to determine optimal dosing regimens and long-term efficacy of metformin in OA management.

Furthermore, the potential impact of metformin on neuropathic aspects of OA should not be overlooked (Zhang et al., 2024; Cao et al., 2024; Pușcașu et al., 2024). As OA can be associated with peripheral neuropathy, particularly in patients with comorbid diabetes or metabolic dysfunction, the ability of metformin to influence glucose metabolism may offer additional therapeutic benefits (Song et al., 2022; Chen et al., 2022; Kaur et al., 2023; Li S. et al., 2024). Previous research has indicated that metformin may reduce nerve damage and improve pain perception in OA patients with diabetes, providing a rationale for its broader application in OA management (Alenazi et al., 2023; Alimoradi et al., 2023; Aiad et al., 2024). Genetic determination plays a crucial role in individual responses to medications (Sydorchuk et al., 2020; Mroziewicz and Tyndale, 2010; Chen et al., 2024). Genetic factors influence the expression and effectiveness of pleiotropic drug effects, including those of metformin, which are being actively studied by various researchers (Buchynskyi et al., 2024a; Pawlyk et al., 2014; Froldi, 2024; Buchynskyi et al., 2024b; Lyubomirskaya et al., 2020).

Finally, while metformin has shown promise in improving metabolic disturbances and reducing inflammation in OA, its efficacy may be limited in certain patient groups, particularly the elderly or those with renal impairment (Kulkarni et al., 2020; Ala and Ala, 2021; Kloppenburg et al., 2025). Our study highlights the need for careful patient selection and monitoring to avoid potential risks, such as lactic acidosis, in vulnerable populations. Further research is needed to investigate the long-term effects of metformin on joint health, its impact on oxidative stress, lipid metabolism, and inflammatory markers, as well as its potential for combination therapy with other disease-modifying agents for OA.

## 5 Limitations

This study has several limitations that should be considered when interpreting the results. First, the relatively small sample size limits the generalizability of the findings to a broader population of patients with osteoarthritis (OA) and impaired glucose tolerance (IGT). Larger, multicenter studies are required to validate these results and confirm their applicability to different populations. Additionally, the study’s observational nature and the lack of randomization may introduce selection bias, and further randomized controlled trials are needed to better assess the causal effects of metformin on OA symptoms and metabolic outcomes.

Moreover, the study only focused on a specific cohort of patients with both OA and IGT, without considering those with OA and normal glucose tolerance, which limits our understanding of metformin’s potential effects across different metabolic states. The absence of long-term follow-up data also prevents us from fully assessing the sustained impact of metformin on joint health, pain, and inflammation over time.

Another limitation lies in the lack of detailed mechanistic data on how metformin influences the molecular pathways underlying both OA and metabolic dysfunction. Future studies should focus on elucidating these pathways and assessing the long-term effectiveness of metformin in modifying OA progression.

Despite these limitations, the findings provide valuable insights into the potential benefits of metformin for managing OA symptoms and metabolic dysfunction, and future research is needed to explore its broader application and long-term impact.

## 6 Conclusion

Patients receiving metformin showed significantly greater improvements in both clinical and metabolic outcomes compared to those not receiving metformin. The metformin group demonstrated reductions in pain, stiffness, and improved physical function, as measured by the WOMAC, Lequesne Algofunctional Index, KOOS, and VAS scales, with notable improvements in quality of life and mobility. In contrast, the non-metformin group showed less significant changes. Metformin also led to reduced inflammatory markers, including C-reactive protein, neutrophil-to-lymphocyte ratio, and superoxide dismutase, suggesting decreased systemic inflammation. Additionally, improvements in lipid profiles, such as reductions in total cholesterol, LDL, HDL, and triglycerides, were observed, highlighting metformin’s metabolic benefits. Patients on metformin also showed significant improvements in BMI, waist-to-hip ratio, and waist-to-height ratio, indicating enhanced metabolic health. The need for increased NSAID doses in patients with osteoarthritis and impaired glucose tolerance can be predicted by factors such as pain severity, functional limitations, and inflammatory markers. Further studies are needed to confirm these findings and assess the long-term effects of dose adjustments. BMI has been identified as a potential predictor of OA exacerbation duration, with a threshold of 29.77. These findings suggest that metformin is effective in alleviating osteoarthritis symptoms and improving metabolic health in patients with osteoarthritis and impaired glucose tolerance. However, further research is needed to explore its long-term effects on joint health and inflammatory markers, as well as its potential role in managing osteoarthritis in patients without impaired glucose tolerance.

## Data Availability

The raw data supporting the conclusions of this article will be made available by the authors, without undue reservation.
